# Molecular phylogeny of Psychodopygina (Diptera, Psychodidae) supporting morphological systematics of this group of vectors of New World tegumentary leishmaniasis

**DOI:** 10.1051/parasite/2023018

**Published:** 2023-05-24

**Authors:** Sonia Zapata, Eunice A.B. Galati, Jaime A. Chaves, Patricio Artigas, Jean-Charles Gantier, Maria Dolores Bargues, Santiago Mas-Coma, Jérôme Depaquit

**Affiliations:** 1 Instituto de Microbiología, Colegio de Ciencias Biológicas y Ambientales, Universidad San Francisco de Quito 170901 Quito Ecuador; 2 Estación de Biodiversidad Tiputini, Colegio de Ciencias Biológicas y Ambientales, Universidad San Francisco de Quito USFQ 170901 Quito Ecuador; 3 Departamento de Epidemiologia, Faculdade de Saúde Pública, Universidade de São Paulo SP 01246-904 São Paulo Brazil; 4 Colegio de Ciencias Biológicas y Ambientales, Campus Cumbayá, Universidad San Francisco de Quito, Diego de Robles y Av. Interoceánica Cumbayá 170901 Quito Ecuador; 5 Department of Biology, San Francisco State University 1600 Holloway Ave San Francisco CA 94132 USA; 6 Departamento de Parasitología, Facultad de Farmacia, Universidad de Valencia, Burjassot Av. Vicente Andrés Estellés s/n 46100 Burjassot Valencia Spain; 7 CIBER de Enfermedades Infecciosas, Instituto de Salud Carlos III C/Monforte de Lemos 3–5. Pabellón 11. Planta 0 28029 Madrid Spain; 8 Laboratoire des Identifications Entomologiques et Fongiques 91540 Mennecy France; 9 Université de Reims Champagne-Ardenne, Structure Fédérative de Recherche Cap Santé, EA 7510 Escape, USC ANSES Petard Reims France; 10 Laboratoire de Parasitologie-Mycologie, Pôle Biologie, Centre Hospitalier Universitaire Reims France

**Keywords:** Sandflies, rDNA, mtDNA, Bayesian analysis, Molecular systematics, Cutaneous leishmaniasis

## Abstract

New World sandflies are vectors of leishmaniasis, bartonellosis, and some arboviruses. A classification based on 88 morphological characters was proposed 27 years ago when the New World phlebotomines were organized into two tribes Hertigiini and Phlebotomini. The latter was structured into four subtribes (Brumptomyiina, Sergentomyiina, Lutzomyiina, and Psychodopygina) and 20 genera. The subtribe Psychodopygina, including most of the American vectors of tegumentary *Leishmania* comprises seven genera from which no molecular work has been produced to support this classification. Here, we carried out a molecular phylogeny based on combined sequences (1,334 bp) of two genes: partial 28S rDNA and mtDNA cytochrome *b* from 47 taxa belonging to the Psychodopygina. The Bayesian phylogenetic reconstruction agreed with the classification based on morphological characters, supporting the monophyly of the genera *Psychodopygus* and *Psathyromyia,* whereas *Nyssomyia* and *Trichophoromyia* seemed to be paraphyletic. The paraphylies of the two latter groups were exclusively caused by the doubtful position of the species *Ny. richardwardi*. Our molecular analysis provides additional support to adopt the morphologic classification of Psychodopygina.

## Abstract (fr)

Les phlébotomes du nouveau monde sont des vecteurs de leishmaniose, de bartonellose et de certains arbovirus. Une classification basée sur 88 caractères morphologiques a été proposée il y a 27 ans lorsque les phlébotomes du nouveau monde ont été organisés en deux tribus Hertigiini et Phlebotomini. Cette dernière était structurée en quatre sous-tribus (Brumptomyiina, Sergentomyiina, Lutzomyiina et Psychodopygina) et 20 genres. La sous-tribu des Psychodopygina, qui inclut la plupart des vecteurs américains de la leishmaniose cutanée, comprend sept genres mais aucun travail moléculaire n’a été produit pour soutenir cette classification. Dans cet article, nous avons réalisé une phylogénie moléculaire basée sur des séquences combinées (1334 pb) de deux gènes : ADNr 28S partiel et cytochrome b (ADNmt) chez 47 taxons appartenant aux Psychodopygina. La reconstruction phylogénique bayésienne est en accord avec la classification basée sur les caractères morphologiques, soutenant la monophylie des genres *Psychodopygus* et *Psathyromyia*, alors que *Nyssomyia* et *Trichophoromyia* semblent être paraphylétiques. Les paraphylies des deux derniers groupes sont exclusivement causées par la position douteuse de la seule espèce *Ny. richardwardi*. Notre analyse moléculaire fournit une raison supplémentaire pour adopter la classification morphologique des Psychodopygina.

## Introduction

Leishmaniases are widespread vector-borne diseases caused by about 20 species [32] of the genus *Leishmania*, transmitted by the bite of Phlebotomine sandflies. They exhibit three main clinical forms: visceral (VL), cutaneous (CL), and mucocutaneous (MCL) leishmaniasis, with a global annual incidence estimated to be between 0.7 and 1 million new cases [9]. In the Americas, 15 species of *Leishmania* are known to affect humans [17]. Among these species, only *L. infantum* is responsible of VL in the New World, with an incidence estimated between 4,500 and 6,800 cases per year. The incidence of both CL and MCL is much higher: between 187,200 and 300,000 new cases yearly [9], mostly caused by *L. braziliensis*. Sandfly vectors have been identified for 12 of these 15 species, with the remaining three (*L. venezuelensis*, *L. lindenbergi,* and *L. martiniquensis*) not having proven vectors [2, 14, 29, 32].

Due to long co-evolution [2, 32], there is a strong link between the *Leishmania* species and the groups of sandflies transmitting them. In the Old World, *L. infantum* is mostly transmitted by sandflies belonging to the subgenus *Larroussius*, whereas *L. major* is transmitted by members of the subgenus *Phlebotomus.* In the New World, all the proven vectors of VL belong to Lutzomyiina, whereas most of the vectors of CL and MCL belong to Psychodopygina, especially the genera *Psychodopygus* and *Nyssomyia*, the main vectors of *L. braziliensis* (Table 1).

**Table 1:**
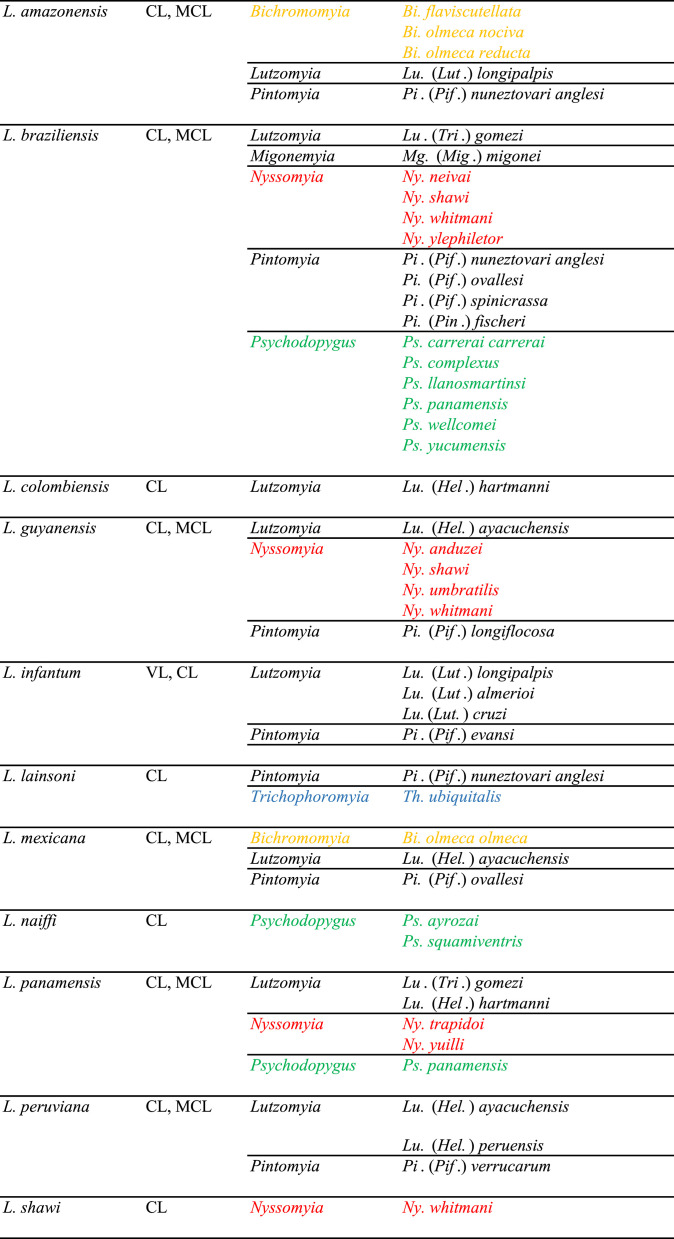
Proven vectors of American *Leishmania* according to Killick-Kendrick (1990), Maroli *et al.* (2013) and Akhoundi *et al.* (2016).

**Figure 1:**
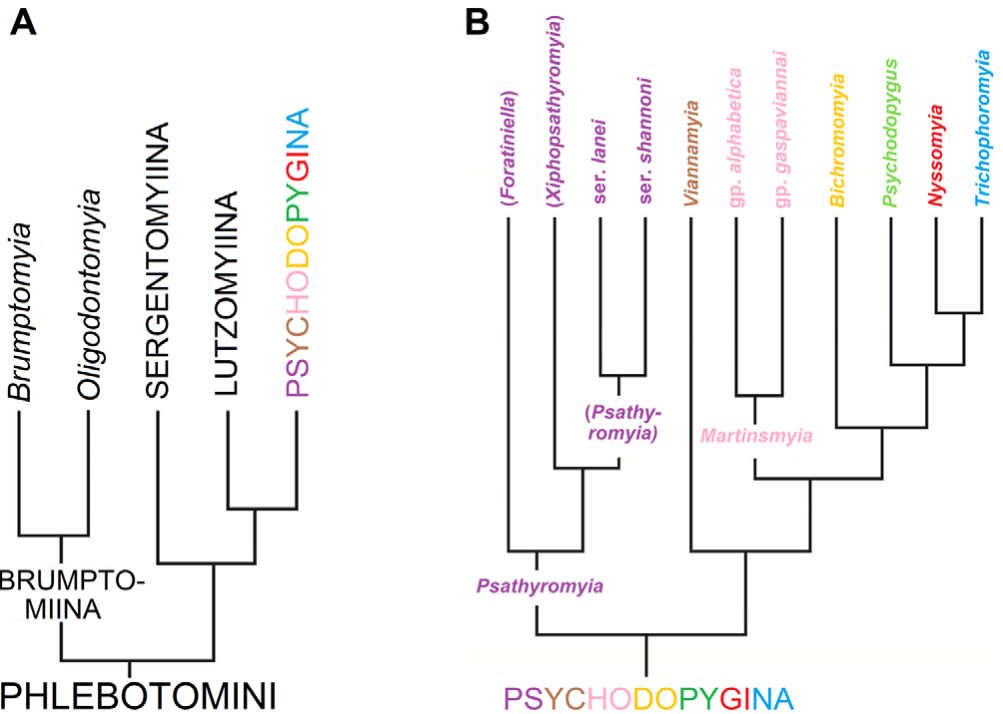
Simplified cladograms based on morphological characters (*sensu* Galati, 1995) representing relationships among Phlebotomini divided into 4 subtribes (A) and among Psychodopygina divided into 7 genera, 3 subgenera, series and groups (B).

The New World Phlebotomine sandflies include more than 500 species distributed throughout North, Central, and South America [25]. Sandfly systematics, established based on morphological characters, were for a long time divided into four genera: *Warileya* Hertig, 1948, *Hertigia* Fairchild, 1949, *Brumptomyia* França & Parrot, 1921, and *Lutzomyia* França, 1924 [47]. A phylogenetic classification, also based on morphological characters was proposed in 1995 by Galati [22], which has since been adopted by most medical entomologists and epidemiologists. It divides the Phlebotomine sandflies into two tribes, Hertigiini Abonnenc & Léger, 1976 and Phlebotomini Rondani & Berte, 1840 [23, 25]. The Hertigiini is a small tribe that includes only nine New World species [4]. It has no proven role in the transmission of *Leishmania* despite the discovery of DNA corresponding to *Leishmania* (*Viannia*) sp. in the anthropophilic *Warileya rotundipennis* [35]. On the other hand, the Phlebotomini tribe includes all the New World vectors. It corresponds to approximately 500 species divided into four subtribes and 20 genera (Fig. 1): i) Brumptomyiina (*Brumptomyia*, *Oligodontomyia* Galati, 1995), ii) Sergentomyiina *partim* (*Deanemyia* Galati, 1995 and *Micropygomyia* Barretto, 1962)*,* iii) Lutzomyiina (*Sciopemyia* Barretto, 1962, *Lutzomyia*, França, 1924, *Migonemyia* Galati, 1995, *Pintomyia* Costa Lima, 1932, *Dampfomyia* Addis, 1945; *Expapillata* Galati, 1995, *Pressatia* Mangabeira, 1942, *Trichopygomyia* Barretto, 1962 and *Evandromyia* Mangabeira, 1941), and iv) Psychodopygina (*Psathyromyia* Barretto, 1962, *Viannamyia,* Mangabeira 1941, *Martinsmyia* Galati, 1995, *Bichromomyia* Galati, 2003, *Psychodopygus* Mangabeira, 1941, *Nyssomyia* Barretto, 1962 and *Trichophoromyia* Barretto, 1962). Although a large number of molecular studies have been published on these groups, the main focus has been to compare closely related species [11]. Regarding the studies including species belonging to different genera, only three have been published to infer phylogenetic relationships between genera of Phlebotomine sandflies [3, 7, 13].

Here we provide for the first time a molecular phylogeny of the Psychodopygina subtribe to compare it with the current morphological classification in use. This work allows for a better understanding of the evolutionary relationships within this group.

## Material and methods

### Sandfly collection

Sandflies were captured using CDC miniature light traps in Mexico, Nicaragua, French Guiana, Ecuador, Brazil, and Bolivia. A total of 48 taxa representing six genera of the Psychodopygina (*Psychodopygus, Nyssomyia, Trichophoromyia, Psathyromyia, Bichromomyia,* and *Martinsmyia*) and one species of the genus *Evandromyia* as an outgroup representative species of the Lutzomyiina subtribe were analyzed (Table 2).

**Table 2:**
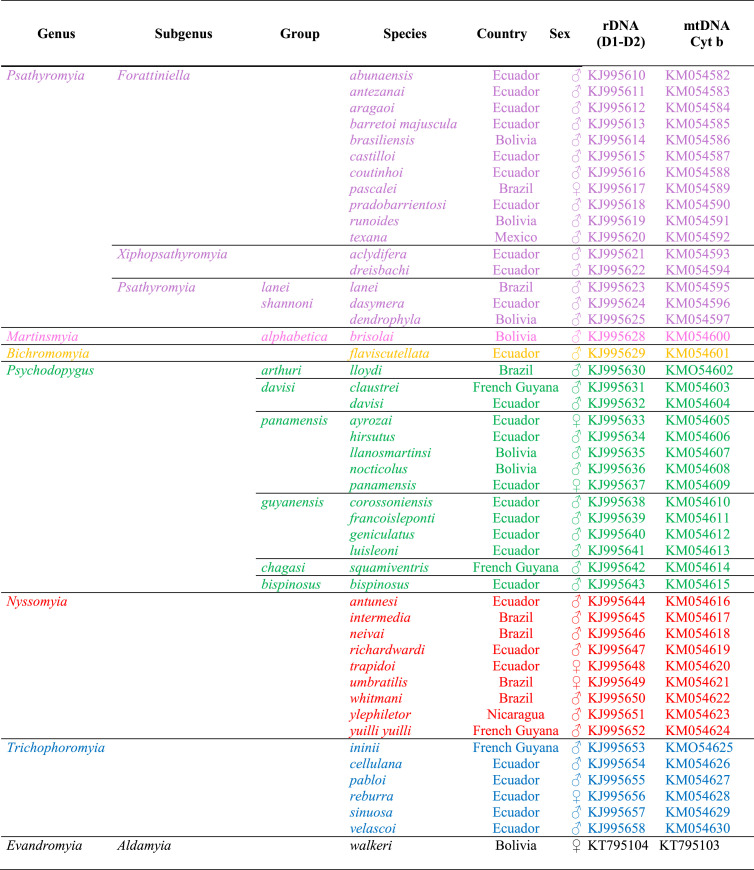
List of specimens analyzed in this study, with country of collection, sex, and GenBank sequence accession numbers.

Head, wings, and genitalia from specimens were cleared and mounted between a slide and cover slide [1]. The thorax related to each specimen was stored at −20 °C or in 96% ethanol before DNA extraction. Some specimens were dried and stored at −20 °C or room temperature waiting for processing. Routinely, two specimens were processed for each taxon, except for scarce species for which only one specimen was processed, and for *Ny. richardwardi* (four specimens processed).

### DNA extraction, PCR amplification, and sequencing

DNA was extracted using a QIAmp^®^ DNA Mini Kit (Qiagen, Hilden, Germany), following a classical protocol [12]. Polymerase chain reactions (PCR) were performed in a 50 μL volume using 5 μL of DNA extracted solution and 50 pmol of primers C3B-PDR/NIN-PDR [18], and C1’/D2 [41] described previously to amplify partial cytochrome *b* (cyt*b*) from mitochondrial DNA and domains D1–D2 from ribosomal DNA 28S, respectively. PCR products were sequenced in both directions using the primers for DNA amplification. Sequences obtained are available in GenBank and accession numbers are indicated in Table 2.

### Sequence alignment and phylogenetic analyses

Sequences were edited and aligned using Muscle software [16] and then manually checked for compliance with the criteria: (1) minimizing the number of inferred mutations; (2) favoring substitutions over insertions and deletions; and (3) favoring transitions over transversions due to the higher probability of their occurrence.

The TVM+I+G model of molecular evolution was determined for both data partitions out of 88 possible models with JModeltest v0.1.0. [9, 39] via the Akaike Information Criterion (AIC) [8]. The AIC values for these models in JModeltest were 7062.889520 for cytb and 11236.768300 for D12, both with a delta AIC of zero. Phylogenetic reconstruction was conducted using Bayesian (BA) inference in MrBayes [43]. Bayesian analyses on the combined two-genes dataset were performed using a mixed model with a partition by gene assigning independent models of evolution to each partition. All parameters were unlinked between partitions, except topology and branch lengths. Analyses consisted of two runs of four simultaneous Markov chains each for 10 million generations, sampling a tree every 1,000 generations and applying a 25% burn-in after checking for convergence using TRACER v1.4 [40] and AWTY [36] to confirm that the standard deviation of split frequencies approached zero. The resulting trees were kept, calculating posterior probabilities in a 50% majority-rule consensus tree. The species *Evandromyia* (*Aldamyia*) *walkeri,* a taxon belonging to the Lutzomyiina, was used as outgroup in our analyses because this subtribe is the sister group of Psychodopygina (Fig. 1).

## Results

### Sequencing

Sequence fragments for D1 and D2 amplified segments, including the flanking conserved domains of *rDNA28S* were 687 to 690 base pairs (bp). Their alignment included 690 nucleotide positions: 535 constant, 153 variable but parsimony uninformative, and 86 were parsimony informative. The lengths of partial cytochrome *b* sequences obtained ranged from 501 to 545 bp. The final alignment dataset included 545 nucleotide positions: 288 were constant, 235 were variable but parsimony uninformative, and 176 were parsimony informative. No intraspecific variation was observed for both markers.

### Phylogenetic reconstruction

**Figure 2:**
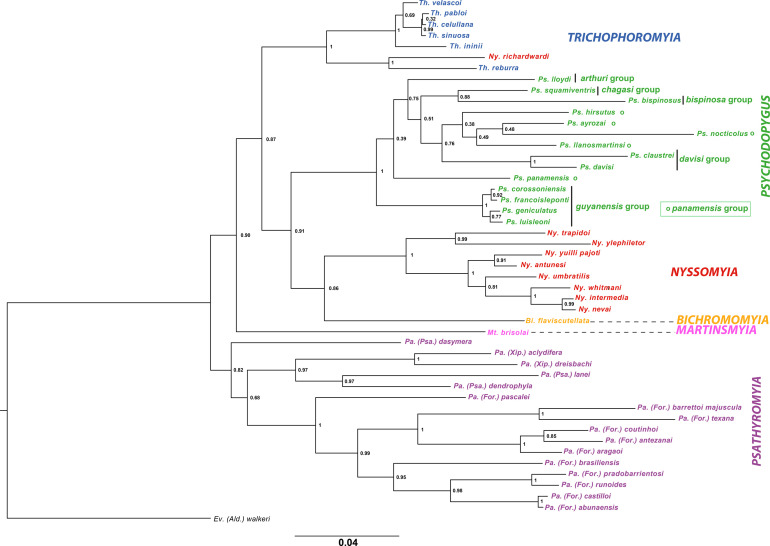
Phylogenetic reconstruction of the Psychodopygina subtribe from Bayesian analysis based on the sequences of D1 and D2 domains of 28SrDNA and cytochrome *b* gene of mtDNA. The highlighted groups and dotted lines correspond to the six genera analyzed. The numbers in the nodes represent posterior probabilities.

**Figure S1:**
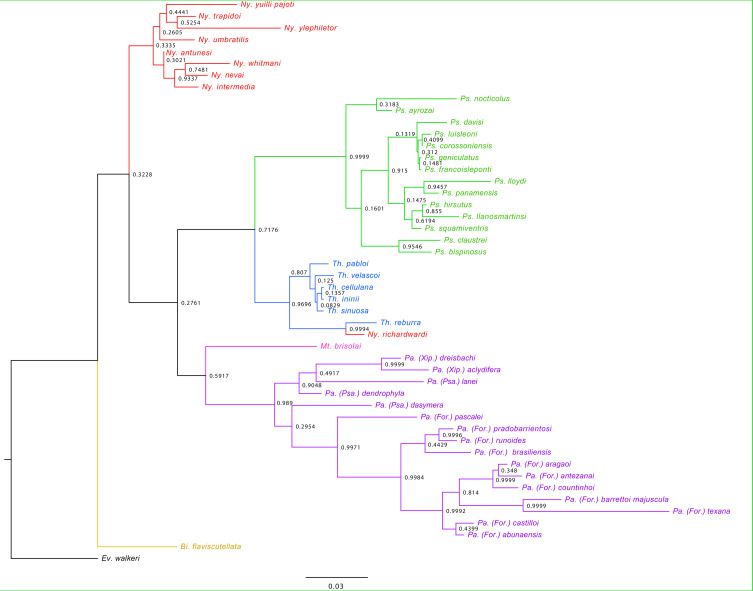
Phylogenetic reconstruction of the Psychodopygina subtribe from Bayesian analysis based on sequences of the D1 and D2 domains of 28SrDNA, the numbers in the nodes representing posterior probabilities.

**Figure S2:**
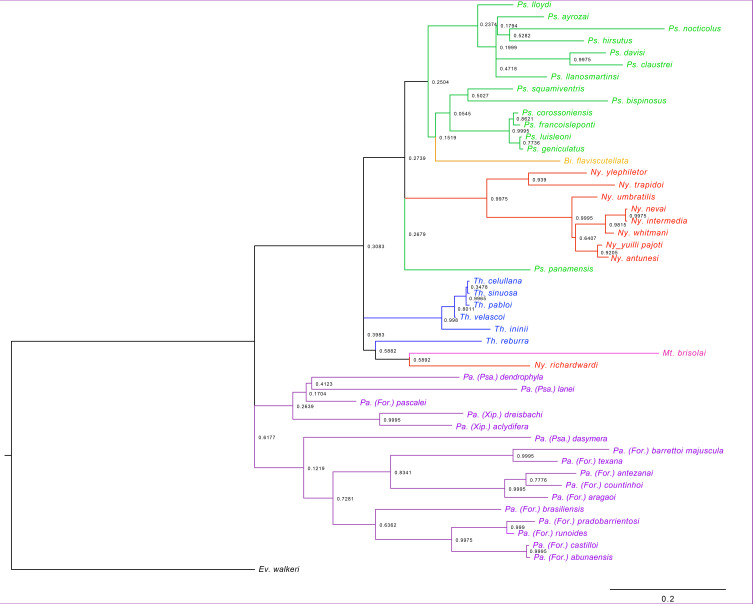
Phylogenetic reconstruction of the Psychodopygina subtribe from Bayesian analysis based on sequences of the cytochrome *b* gene of mtDNA, the numbers in the nodes representing posterior probabilities.

The Bayesian analysis of the combined two-gene dataset (1,334 bp) recovered highly supported monophyletic groups with posterior probabilities (PP > 0.9) for most of the clades (Fig. 2). Some of these same clades were not recovered using both genes independently as nodal support was not strong (PP < 70) (Supplementary Figures S1 and S2).

The recovered phylogenetic reconstruction supports the monophyly of the genus *Psathyromyi*a, sister to all other Psychodopygina members. The genus *Psathyromyi*a was composed of three subgenera: *Forattiniella, Psathyromyia,* and *Xiphopsathyromyia*. The subgenera *Forattiniella* and *Xiphopsathyromyia* were found to be monophyletic, whereas the subgenus *Psathyromyia* appeared as paraphyletic.

The monophyly of the genus *Martinsmyia* was not confirmed because we processed only one species. Moreover, the genus *Martinsmyia* was composed of the groups *alphabetica* (here represented by *Mt. brisolai*) and *gasparviannai* from which no sample was analyzed. The genus *Trichophoromyia* was recovered as paraphyletic because it included one species belonging to the genus *Nyssomyia* (*Ny. richardwardi*) (Fig. 2), rendering the genus *Nyssomyia* also paraphyletic. The position of *Ny. richardwardi* varied depending on the selected marker. According to D1D2 28S rDNA, it was recovered within the genus *Trichophoromyia*, sister to *Th. reburra*. On the other hand, the analysis of cyt *b* placed this species sister to *Mt. brisolai* (Supplementary Figures S1 and S2). If we excluded *Ny. richardwardi* from the combined dataset as well as from each dataset independently (cytb or rDNA), the genera *Trichophoromyia and Nyssomyia* were recovered monophyletic with high confidence (PP > 0.9).

The genus *Psychodopygus* was found to be monophyletic and divided into six groups. The group *guyanensis*, represented here by 4 of its 7 species (*Ps. corossoniensis*, *Ps. luisleoni*, *Ps. francoisleponti,* and *Ps. geniculatus*) and the group *davisi* (*Ps. davisi* and *Ps. claustrei*) appeared monophyletic with high nodal support (Fig. 2). The rest of the group had lower posterior probabilities, from which the group *panamensis* was found to be paraphyletic with *Ps. hirsutus*, *Ps. ayrozai*, *Ps. nocticolus*, and *Ps. llanosmartinsi* placed in one clade that excluded the congener *Ps. panamensis*. Furthermore, the groups *chagasi*, *arthuri*, and *bispinosus* were represented by only one species (*Ps. squamiventris squamiventris, Ps. lloydi* and *Ps. bispinosus*, respectively) and their position was less supported.

## Discussion

To our knowledge, there is only one morphological phylogeny study, apart from the studies by Galati [22], that focus on the subgenus *Lutzomyia* in New World sandflies [38]. However, a large number of molecular studies related to Phlebotomine sandflies have been carried out [11], but mostly focused on closely related species. On the other hand, few studies concerning the deep phylogeny of Phlebotomine sandflies, that is comparing several genera, have been published. For instance, the work by Depaquit *et al.* [13] and Aransay *et al.* [3] focused primarily on Old World sandflies and only included a few New World species. The most comprehensive analysis and the one that included the largest number of taxa is that of Grace-Lema *et al.* [27]. But despite the extension of several loci, this work suffered from a lack of data that could explain the dubious position of the genus *Brumptomyia* or the paraphyly of the genus *Nyssomyia*. Most molecular studies on New World sandflies have focused either on several closely related species of the same genus or subgenus [6, 10, 44, 46] or on the population level (i.e. population genetics) [19, 28, 37, 45]. From the eight publications available to date based on several genera of New World sandflies (Table 3), only the work by Beati *et al.* (2004) combined cyt*b* with partial 28S rDNA sequences [7]. The rest of these phylogenies are exclusively based on one mitochondrial marker (cyt*b*, COI, 12S or 16S) limiting alternative phylogenetic exploration. Consequently, Beati *et al.* [7] mostly focusing on Lutzomyiina from Andean regions partially tested Galati’s hypotheses. Here we summarize some of the results in light of their findings and to contrast with our proposed phylogeny. Dutari & Loaiza [15] focused primarily on sandflies from Panama recovering *Pi. ovallesi* and *Mi. trinidadensis* as part of the Psychodopygina clade. This position is probably artefactual due most likely to the effect of long-branch attraction. The study by Kocher *et al.* [30], based on 16S mtDNA of 40 species of Lutzomyiina, Psychodopygina, and Brumptomyiina exclusively from French Guiana, revealed that *Ny. sylvicola* is distantly related to the other *Nyssomyia* lineages. This conflicting result could be most likely related to the resolution of this marker, not ideal at species-level phylogenies. Next, Cohnstaedt *et al.* [10] based its phylogeny on cyt*b* marker and 10 species of *Pintomyia* (*Pifanomyia*) belonging to the groups *verrucarum*, *serrana*, and *townsendi*, produced a non-surprising phylogeny considering the analyzed species were closely related ones.

**Table 3:** Molecular studies carried out on New World Sandflies, comparing several genera.

Study	Gene(s) sequenced	Sampling	Main goal
Torgerson *et al.* (2003)	cyt b mtDNA	12 species of Lutzomyiina and Psychodopygina rooted on two *Brumptomyia*	Phylogeny
Beati *et al.* (2004)	12S mtDNA & D4–D5 of 28SrDNA	30 species mostly Lutzomyiina and a few Psychodopygina	Phylogeny
Cohnstaedt *et al.* (2011)	cyt b mtDNA	10 species of *Pintomyia* (*Pifanomyia*) belonging to the series Verrucarum, Serrana and Townsendi	Phylogeny
Yamamoto *et al.*. (2013)	cyt b mtDNA	12 species of *Lutzomyia* and *Pintomyia* rooted on *Warileya*	Phylogeny
Grace-Lemma *et al.* (2015)	Cac COI, Cyt b and ND4 mtDNA; ITS2 rDNA; cacophony	32 species of New world sandflies	Phylogeny
Romero-Ricardo *et al.* (2016)	COI mtDNA	19 species of Lutzomyiina and Psychodopygina rooted on *Brumptomyia*	DNA barcoding
Kocher *et al.* (2017)	16S mtDNA	40 species of Lutzomyiina, Psychodopygina and Brumptomyiina	Metabarcoding by NGS
Dutari & Loaiza (2019)	COI mtDNA	14 species of Lutzomyiina and Psychodopygina rooted on *Brumptomyia*	DNA barcoding

**Figure 3:**
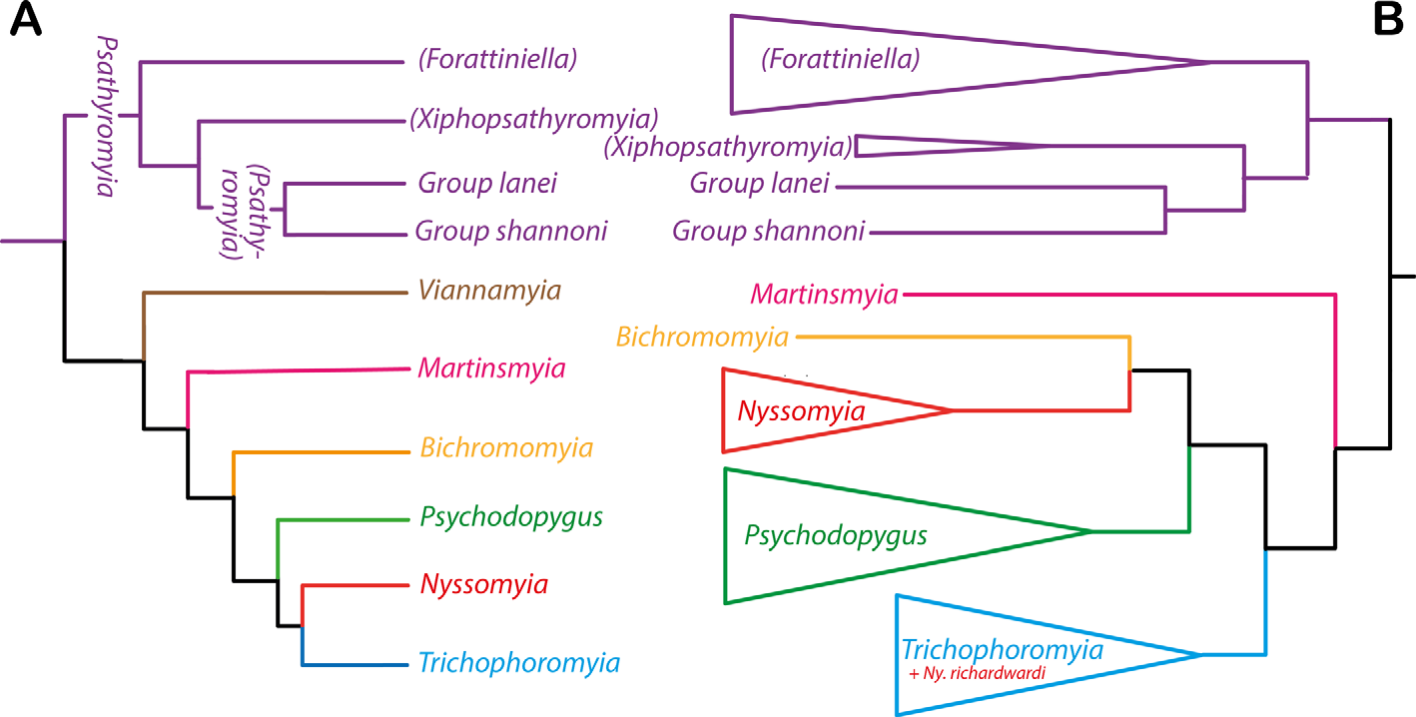
Phylogenetic relationships within the Psychodopygina according to Galati’s hypothesis (A) and according to the molecular data of the present study (B).

*Psychodopygus* was raised for the first time to the genus level by Forattini [20, 21] which at that time, comprised the greatest number of the species now included in the genera *Bichromomyia*, *Psychodopygus, Nyssomyia*, *Trichophoromyia* and *Psathyromyia*, *partim* of Psychodopygina by Galati [22]*.* Our study is strongly in agreement with Galati’s hypotheses (Fig. 3). However, Forattini considered *Viannamyia* as a distinct genus, but Galati 1981’s classification [24] based on the morphology and implantation level of the larvae antennae, included the *Viannamyia* subgenus in *Psychodopygus*. We were not able to test this hypothesis given the lack of samples from *Viannamyia*. On the other hand, Ready *et al.* [42] considered only *Psychodopygus* as a distinct genus from *Lutzomyia,* arguing that the particular spermathecae of the females (with imbricated triangular rings) would justify its differentiation. Our phylogenetic reconstructions strongly support the monophyly of the genus *Psychodopygus sensu* Ready *et al.* [42] and Galati [22] as shown by both the concatenated dataset (ribosomal and mitochondrial, Fig 2.) and by each marker (Supplementary figures). The groups (formerly called series in the literature), *guyanensis* and *davisi* are monophyletic except for the group *panamensis*. This paraphyly is caused by the position of the group *davisi* separating *Ps. panamensis* from the other members of the group.

The genus *Nyssomyia,* considered monophyletic by Galati [23] appears paraphyletic considering the position of *Ny. richardwardi* grouped within the genus *Trichophoromyia*. If we exclude the latter species of *Nyssomyia,* it could be considered as a clade subdivided into two well-supported monophyletic groups: one clade including the related species *Ny. trapidoi* and *Ny. ylephiletor* and a second clade including *Ny. yuilli pajoti*, *Ny. umbratilis* and *Ny. antunesi* grouped with the closely related species *Ny. whitmani, Ny. intermedia* and *Ny. neivai*. Additionally, introgression has been described for *Ny. whitmani* and *Ny. intermedia* [31, 33, 34] and both male and female hybrids between *Ny. intermedia* and *Ny. neivai* were observed [26]. We confirm the phylogenetic position of *Ny. richardwardi* was not due to a sequencing or laboratory error because four specimens were processed and sequenced at different times obtaining 100% identical data each time. Following the morphological identification keys of Galati [23–25], *Ny. richardwardi* and *Ny. shawi* form a cohesive group within the genus *Nyssomyia* with some morphologic characters shared across the genera *Nyssomyia* and *Trichophoromyia* (Table 4). For example, both species present simple setae on all flagellomeres, and in the males, the internal spine of the gonostyle is implanted close to its base. These characteristics are present in *Trichophoromyia* but not in *Nyssomyia*. However, the absence of Newstead sensilla on the palpomere II and spermathecae with less than 20 rings are present in *Nyssomyia* but not in *Trichophoromyia.* Further, both species exhibit cerci longer than wide in females from *Nyssomyia* but different from those of *Trichophoromyia*. In the morphological analysis, the clade constituted by both genera *Nyssomyia* and *Trichophoromyia* share more synapomorphies with *Psychodopygus* than with *Bichromomyia* in Galati’s cladogram (Fig. 1); however, in our phylogenetic tree (Fig. 2) the genus *Bichromomyia* appears as the sister group of *Nyssomyia*.

**Table 4:**
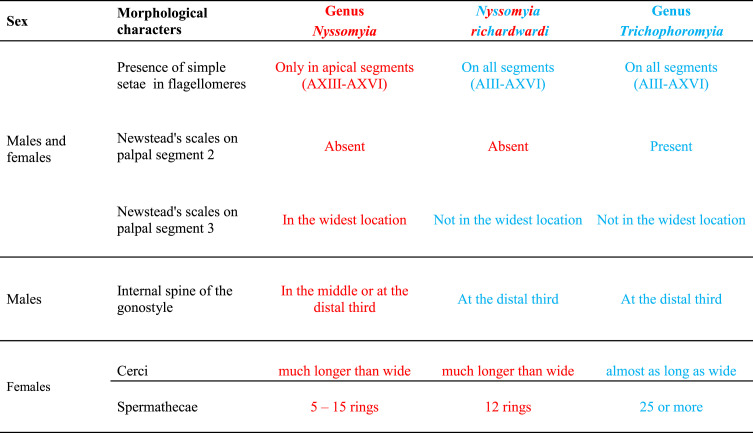
Morphological characters of *Ny. richardwardi* shared with the *Nyssomyia* and *Trichophoromya* genera.

The genus *Psathyromyia* was found to be monophyletic as well as the subgenera *Forattiniella* and *Xiphopsathyromyia*. However, the position of *Pa. dasymera* as sister to all other *Psathyromyia* species, including the members of the groups *shannoni* and *lanei*, renders the genus *Psathyromyia* paraphyletic.

Unfortunately, it was not possible to include samples belonging to the genus *Viannamyia* in our analysis. As a consequence, the phylogenetic position of *Viannamyia* within the Psychodopygina subtribe remains unsolved. In the future, exploring the relationships between the New World *Leishmania* agents of tegumentary leishmaniasis belonging to the genera *Leishmania*, *Viannia,* and *Mundinia* [5] and their vectors will prove to be essential in understanding specific vector associations and disease ecology. We suggest expanding on these phylogenetic explorations to clarify the taxonomic assessment of these vectors, strongly related to human and animal health.

## Supplementary material

The supplementary material of this article is available at https://www.parasite-journal.org/10.1051/parasite/2023018/olm.
